# P2Y1 agonist HIC in combination with androgen receptor inhibitor abiraterone acetate impairs cell growth of prostate cancer

**DOI:** 10.1007/s10495-022-01716-1

**Published:** 2022-02-07

**Authors:** Hien Thi Thu Le, Akshaya Murugesan, Nuno R. Candeias, Thiyagarajan Ramesh, Olli Yli-Harja, Meenakshisundaram Kandhavelu

**Affiliations:** 1 Molecular Signaling Group, Faculty of Medicine and Health Technology, Tampere University and BioMediTech, P.O.Box 553, 33101 Tampere, Finland; 2 Department of Biotechnology, Lady Doak College, Thallakulam, Madurai, 625002 India; 3 Faculty of Engineering and Natural Sciences, Tampere University, Korkeakoulunkatu 8, 33101 Tampere, Finland; 4 LAQV-REQUIMTE, Department of Chemistry, University of Aveiro, 3810-193 Aveiro, Portugal; 5 Department of Basic Medical Sciences, College of Medicine, Prince Sattam Bin Abdulaziz University, Al-Kharj, 11942 Kingdom of Saudi Arabia; 6 Computational Systems Biology Research Group, Faculty of Medicine and Health Technology and BioMediTech, Tampere University, P.O.Box 553, 33101 Tampere, Finland; 7 Institute for Systems Biology, 1441N 34th Street, Seattle, WA 98103-8904 USA

**Keywords:** Prostate cancer, Abiraterone acetate, Indoline, Apoptosis, Proliferation

## Abstract

P2Y receptors belong to the large superfamily of G-protein-coupled receptors and play a crucial role in cell death and survival. P2Y1 receptor has been identified as a marker for prostate cancer (PCa). A previously unveiled selective P2Y1 receptor agonist, the indoline-derived HIC (1-(1-((2-hydroxy-5-nitrophenyl)(4-hydroxyphenyl)methyl)indoline-4-carbonitrile), induces a series of molecular and biological responses in PCa cells PC3 and DU145, but minimal toxicity to normal cells. Here, we evaluated the combinatorial effect of HIC with abiraterone acetate (AA) targeted on androgen receptor (AR) on the inhibition of PCa cells. Here, the presence of HIC and AA significantly inhibited cell proliferation of PC3 and DU145 cells with time-dependent manner as a synerfistic combination. Moreover, it was also shown that the anticancer and antimetastasis effects of the combinratorial drugs were noticed through a decrease in colony-forming ability, cell migration, and cell invasion. In addition, the HIC + AA induced apoptotic population of PCa cells as well as cell cycle arrest in G1 progression phase. In summary, these studies show that the combination of P2Y1 receptor agonist, HIC and AR inhibitor, AA, effectively improved the antitumor activity of each drug. Thus, the combinatorial model of HIC and AA should be a novel and promising therapeutic strategy for treating prostate cancer.

**Supplementary Information:**

The online version contains supplementary material available at 10.1007/s10495-022-01716-1.

## Introduction

Prostate cancer (PCa) is the most common malignant tumour diagnosed in men around the world [1]. It is known as the third leading cancer causing death in males, estimated at around 0.3 million patients’ death per year [2]. However, metastatic PCa remains incurable due to its resistant characteristics against many chemotherapeutic drugs. Recently, second-generation anti-androgen therapy such as abiraterone acetate (AA), apalutamide, bicalutamide, etc. [3–7] is considered as a potential therapy for treating PCa. The research for novel strategies and regimens to treat PCa has being continued but reducing the doses of drugs and improving the patients’ lives remains an open challenge.

Anti-androgen therapy is often utilized to treat PCa and increase the patients’ survival [8, 9]. In the early stage of PCa, medical or surgical castration is the common method for patients treatment [10, 11]. However, the disease can still progress and be resistant to the primary treatment. Such a type of cancer is called castrate-resistant PCa (CRPC) [12]. In androgen receptor (AR)-positive cell line (LNCap cells), the anticancer effect of androgen biosynthesis inhibitor AA was observed to occur via the degradation of the CYP17A1 enzyme. This cytochrome P450, family 17, subfamily A, polypeptide 1 enzyme is crucial for androgen-dependent cancers and hyperplasia [13, 14]. AA inhibits the production of androgens by interfering with the enzymes C17α hydroxylase and C17-C20 lyase, which suppresses PCa cells growth and metastasis [15]. Based on the down-regulated mechanism of AA, the common hypothesis is that the drug reduces the expression of AR and inhibits AR signaling, which is the crucial antitumor activity of the drug [16]. The clinical studies reported promising results in Phase I and II trials of patients with metastatic CRPC tested [17, 18]. Thus, AA (Zytiga, Janssen Biotech Inc.) was accepted by the US Food and Drug Administration (FDA) for patients with metastatic CRPC who had received prior chemotherapy [19]. FDA later approved the use of AA tablets in combination with castration and prednisone for the treatment of metastatic high-risk castration sensitive PCa (CSPC) [20, 21]. We have previously found limited evidence of AA activity, at low concentrations of AA, in inhibiting the proliferation of AR-negative PC3 and DU145 cells [22, 23]. Martina et al. reported that 2 μM of AA did not show cell death in PC3 and DU145 after 96 h treatment [22]. Additionally, it is evident that treatment of 30 μM AA can induce apoptosis on PC3 cell death [23]. On the other hand, purinergic receptor 1 (P2Y_1_R) is highly expressed in PC3 and DU145 cells in both normal and cancer cells [24–27]. The activation of P2Y_1_R is suggested as a therapeutic target for suppressing PCa cell growth [26]. For example, MRS 2365, a selective agonist of P2Y1R, decrease cell proliferation and increase apoptotic cells’ and Caspase 3’s activities in PC3 cells [26]. Recently, we designed and synthesized a P2Y_1_R agonist, 1-(1-((2-hydroxy-5-nitrophenyl)(4-hydroxyphenyl)methyl)indoline-4-carbonitrile (HIC) to activate the P2Y_1_R signaling [28–30]. HIC is a time- and dose-dependent selective inhibitor of PC3 and DU145 cell growth [28]. In addition, the activation of P2Y1R induces apoptosis, Caspase 3/7 activity, and ROS production in these cell lines [28]. Although the activity of HIC was identified as a potential drug-like compound again the growth of PC3 and DU145, the combinatorial effect of HIC along with any known clinical drug is yet to be investigated.

In this work, we aim to investigate the combinatorial effect of HIC and AA in the treatment of PC3 and DU145 cells. Here we measure the PCa cell death in a dose- and time-dependent manner, and colony formation. We have also measured the combinational index (CI) for the combinatorial activity of HIC and AA. Further, the anticancer and anti-metastasis effects were evaluated by apoptosis, caspase 3/7, ROS formulation, wound healing, and invasion assay. Finally, we have also measured the combinatorial effect of HIC and AA on PCa cell phase arrest. The comprehensive in vitro evaluation of combined HIC and AA of the present study will provide a basis for future clinical studies.

## Materials and methods

### Preparation of chemicals

Compound 1-(1-((2-hydroxy-5-nitrophenyl)(4-hydroxyphenyl)methyl)indoline-4-carbonitrile (HIC) was synthesized as described previously [28]. Abiraterone acetate (AA) was purchased from Sigma-Aldrich (St. Louis, MO, USA). HIC and AA compounds were diluted at 100 μM stocks in dimethyl sulphoxide (DMSO; Sigma-Aldrich).

### Cell culture

PCa cell lines, PC3 and DU145, were cultured in Minimum essential medium eagle (MEME; Sigma-Aldrich). Non-cancer cell lines HEK293 and MEF were maintained in Dulbecco’s modified eagle medium–high glucose (DMEM; Sigma-Aldrich). Mediums were supplemented with 10% Fetal bovine serum (Biowest, Nuaille, France), 0.1 mg/mL streptomycin, 100 U/mL penicillin (Sigma-Aldrich), and 0.025 mg/mL amphotericin B (Sigma-Aldrich). Cells were grown at 37 °C in a humidified condition of 5% CO_2_. Cells were passaged every 3–4 days using trypsin 1X (Sigma-Aldrich). To prepare cells for assays, cells were counted using trypan blue solution (Sigma-Aldrich) and countess II FL automated cell counter (ThermoFisher Scientific, Waltham, MA, USA).

### Cell proliferation and cytotoxicity assay

To determine the sensitivity of PCa and noncancer cells to HIC and AA, cells were plated with a density of 1 × 10^4^ cells/well in 96-well clear-bottom plates for 24 h. Cells were dosed with various concentrations of HIC (1.25, 2.5, 5, 7.5, 15, and 40 μM) and AA (5, 10, 20, 30, 40, and 50 μM) for 48 h. For the combinational drug model, PCa cell lines and noncancer cells were seeded in 96-well clear-bottom plates at a density of 1 × 10^4^ cells/well for 24 h incubation. PC3 and DU145 cells were incubated with DMSO, HIC (1, 5, 10, 15, and 20 μM), AA (3, 15, 30, 45, and 60 μM), or combination of HIC and AA for 48 h. HEK293 and MEF cells were treated with HIC (1, 5, 10, and 15 μM) and AA (3, 15, 30, and 45 μM) together. After 48 h treatment, cell death was determined using MTT cell proliferation and cytotoxicity assay kit (Bosterbio, CA, USA) as described previously [28]. Briefly, the cells were labelled with MTT labelling reagent (0.5 mg/mL MTT reagent final concentration in phosphate-buffered saline, PBS) and incubated for 4 h in a humidified chamber. Then, formazan solubilization solution was added to each well and kept for 4 h in a dark condition. Treatments were carried out in triplicate. The optical densities (OD) of the supernatants were measured at 570 nm using a Magellan™ microplate reader (Tecan Group Ltd., Switzerland). The inhibitory effects of drugs were calculated using the equation given below,

1 $$ \% inhibition = \frac{{A_{c} - A_{tr} }}{{A_{c} }} \times 100 $$

where *A*_*c*_ is the cell number of untreated cells, and *A*_*tr*_ is the cell number of drugs treated cells. DMSO treated groups was considered as the vehicle control.

### Combination therapy assays

The mechanism of drug interaction was determined using the combination index (CI). CI values were calculated using the CompuSyn software (ComboSyn Inc., Paramus, NJ, USA) following the below equation [31, 32].

2 $$ CI = \frac{{\left( {D1} \right)}}{{\left( {Dx} \right)1}} + \frac{{\left( {D2} \right)}}{{\left( {Dx} \right)2}} $$

(Dx)1 and (Dy)2 are the concentration of each drug required to produce the same effects as the effect produced by doses D1 and D2 in the combination [(D1) + (D2)]. CI values < 0.9, 0.9 < CI < 1.1, and 1.1 < CI indicate synergistic, additive, and antagonistic effect of two drugs, respectively [33].

### Pharmacokinetic assay

PC3 and DU145 cells were seeded in a 96-well plate at 1 × 10^4^ cells/well. After 24 h incubation, PCa cells were treated with DMSO, 10 μM HIC, 30 μM AA, and 10 μM HIC + 30 μM AA for 24, 48, and 72 h in an incubator. MTT cell proliferation and cytotoxicity assay kit were used to determine cell death. Inhibition percentage was analyzed using Eq. 1 for cell viability assay. DMSO sample was used as vehicle control.

### Colony assay

PC3 and DU145 cells were seeded at 500 cells per well in six-well plates and incubated overnight. The cells were then treated with DMSO, 10 μM HIC, 30 μM AA, or with combinations of HIC and AA. The media with and without drugs were refreshed every 3 days. After incubation for 12 days, the plates were washed gently two times with PBS. Colonies were then treated with fixing solution (3.7% paraformaldehyde in PBS) for 10 min in RT. The colonies were washed two times with PBS and stained with a 0.05% crystal violet solution for 10 min at room temperature. Images were captured under a microscope. Colonies over 50 cells were counted directly using an Axiovert 200 M microscope (Carl Zeiss, Germany). Survival fraction was measured using Eq. 3. DMSO-treated samples were considered vehicle samples.

3 $$ Inhibitory\, ratio \left( \% \right) = \frac{No.\, of \,colonies\, treated \,with\, drugs}{{No.\,of\, cell\, treated\, with\, vehicle}} \times 100\% $$

### 4’,6-Diamidino-2-Phenylindole, dihydrochloride (DAPI), Annexin V, and propidium iodide (PI) staining assay

To determine the induction of apoptotic and necrosis by that target drugs, we carried out the cell apoptosis assay using a dead cell apoptosis kit (Sigma-Aldrich) and DAPI staining (ThermoFisher Scientific). PC3 and DU145 cells were plated in a six-well plate with a density of 5 × 10^5^ cells/well. After 24 h incubation, cells were incubated with DMSO, 10 μM HIC, and/or 30 μM AA for 48 h. The cells were collected with PBS and then incubated with 50 μL 1X Annexin-binding buffer from the kit for 15 min in the dark condition. Then 5 μL FITC conjugated Annexin V, 1 ng/mL PI, and 300 nM DAPI was added to the cell suspension for 15 min in an incubator. The fluorescence images of cells were captured using EVOS FL (ThermoFisher Scientific) under 20 × objective for each analysis. The fluorescence microscopy image data was analyzed to find out the differences in the plasma membrane integrity and permeability using Annexin V/PI dual staining. We have integrated both the semi-automated image processing along with manual counting to extract the fluorescence intensity values and thus measured the percentage of apoptotic and necrotic cells.

### Caspase 3/7 activity

To determine the caspase 3/7 activity, PC3 and DU145 cells were seeded in 96-well white plates at a density of 1 × 10^4^ cells/well overnight. The cells were treated with DMSO, 10 μM HIC, 30 μM AA, and combinational 10 μM HIC and 30 μM AA for 5 h. AA is the suitable control which not only act as a anti-androgen therapy for treating prostate cancer, but also functions as a caspase-3 inhibitor. Caspase 3/7 was measured by using a Caspase-Glo®3/7 assay kit (Promega, Madison, WI, USA) following the manufactural protocol. Caspase-Glo reagent was added to the cells and then incubated in an incubator for 1 h. The luminescence of the samples was measured using a Magellan™ microplate reader. The fold change of Caspase 3/7 activity was determined using the below equation,

4 $$ Fold \,increase = \frac{{L_{test} - L_{blank} }}{{L_{control} - L_{blank} }} $$

where: *L*_*test*_ is the luminescence of drugs treated wells; *L*_*blank*_ is the luminescence of untreated wells and; *L*_*control*_ is the luminescence of the unstained wells.

### ROS assay

PC3 and DU145 cells were plated in 12-well plates with a density of 1 × 10^5^ cells/well overnight. The cells were treated with 10 μM HIC, 30 μM AA, 10 μM HIC + 30 μM AA, and 10 mM hydrogen peroxide (H_2_O_2_) as a positive control for ROS for 5 h. The cells were collected and then incubated with 20 μM 2’,7’-dichlorodihydrofluorescein diacetate (H2DCFA) (Sigma-Aldrich) for 30 min in the dark condition. The stained cells were washed 2 times with PBS and incubated in the culture medium for 20 min in an incubator. The fluorescence of ROS products was measured at 485 nm (excitation) and 538 nm (emission) by a Magellan™ microplate reader. The fold change of ROS production was calculated according to Eq. 5.

5 $$ Fold \,change = \frac{{F_{test} - F_{blank} }}{{F_{control} - F_{blank} }} $$

where: *F*_*test*_ is the fluorescence of drugs treated cells; *F*_*control*_ is the fluorescence of untreated cells and; *F*_*blank*_ is the fluorescence of unstained cells.

### Cell cycle analysis

PC3 and DU145 cells were plated in a 6-well plate at the density of 5 × 10^6^ cells/well. After 24 h incubation, the cells were dosed with DMSO, 10 μM HIC, 30 μM AA, or the combination of HIC and AA for 48 h. Cell phases were determined using a propidium iodide kit (Sigma-Alrich). The cells were washed two times with cold PBS and then fixed in 70% ethanol on ice for 10 min. Subsequently, the cells were dyed in 200 μL of PI-Triton-RNase solution (20 μg/mL PI, 0.2 mg/mL RNase, and 0.1% Triton X-100 in PBS) for 15 min in the dark condition. Fluorescence images were captured using EVOS FL at 20 × magnification. The images were analyzed using CellProfiler software 4.0 [34] and cell cycle phases were calculated using MATLAB R2020a (MathWorks Ltd., MA, USA).

### Wound-healing assay

PC3 and DU145 cells were seeded in six-well plates with a density of 1.5 × 10^6^ cells/well for 24 h. At 80–90% confluence, the wound was made with 200 μL pipette tips. The floating cells were removed by washing the plates two times with warm PBS. The cells were then incubated with DMSO, 10 μM HIC, and/or 30 μM AA in 1% FBS media for 24 h. The images of the migrated cells into the wound surface were captured using EVOS imaging systems. The wound areas were measured using ImageJ software 1.52 (National Institutes of Health, USA). The wound closure was calculated using Eq. 6.

6 $$ W_{change} = \frac{{W_{0 - D} - W_{24 - D} }}{{W_{0 - C} - W_{24 - C} }} \times 100\% $$

where: *W*_*0-D*_ is the scratch area in samples treated with drugs at the starting point; *W*_*24-D*_ is the scratch area in samples treated with drugs after 24 h incubation; *W*_*0-C*_ is the scratch area in vehicle groups at the starting point and; *W*_*24-C*_ is the scratch area in vehicle groups after 24 h incubation. DMSO treated groups were considered as the vehicle groups.

### Invasion assay

Transwells (6-well type, pore size 8 μm) were coated with Corning® Matrigel® Basement Membrane Matrix (Corning, NY, USA) for 2 h in an incubator. PC3 and DU145 cells (5 × 10^5^ cells/well) were seeded to the upper chamber in the absence or presence of HIC and AA in 1% FBS media. The lower chamber was completely filled with FBS medium. After 24 h incubation, the chambers’ membranes were treated with Fixing solution (3.7% paraformaldehyde in PBS) for 10 min. Next, the chambers were washed gently with warm PBS. The membranes with invaded cells were stained with 0.5% crystal violet in 2% EtOH. After 5 min, the membranes were washed with PBS and kept dry at room temperature. Five random fields of the membranes were observed. Invaded cells were calculated based on the average number of cells from five random areas. Data were presented as the percentage of the number of invaded cells based on the vehicle group.

### Statistical analysis

All the experiments were repeated three or five times with the same biological and technical conditions. The results are represented as means ± standard error of the mean (SEM) using IBM SPSS Statistics version 26. Differences between samples groups and experimental conditions were analyzed using one-way ANOVA followed by GraphPad Prism 8.0 software. Statistical significance was considered with *p < *0.05*.

## Results

### Sensitivity of PCa and noncancer cells to HIC and AA

**Fig. 1:**
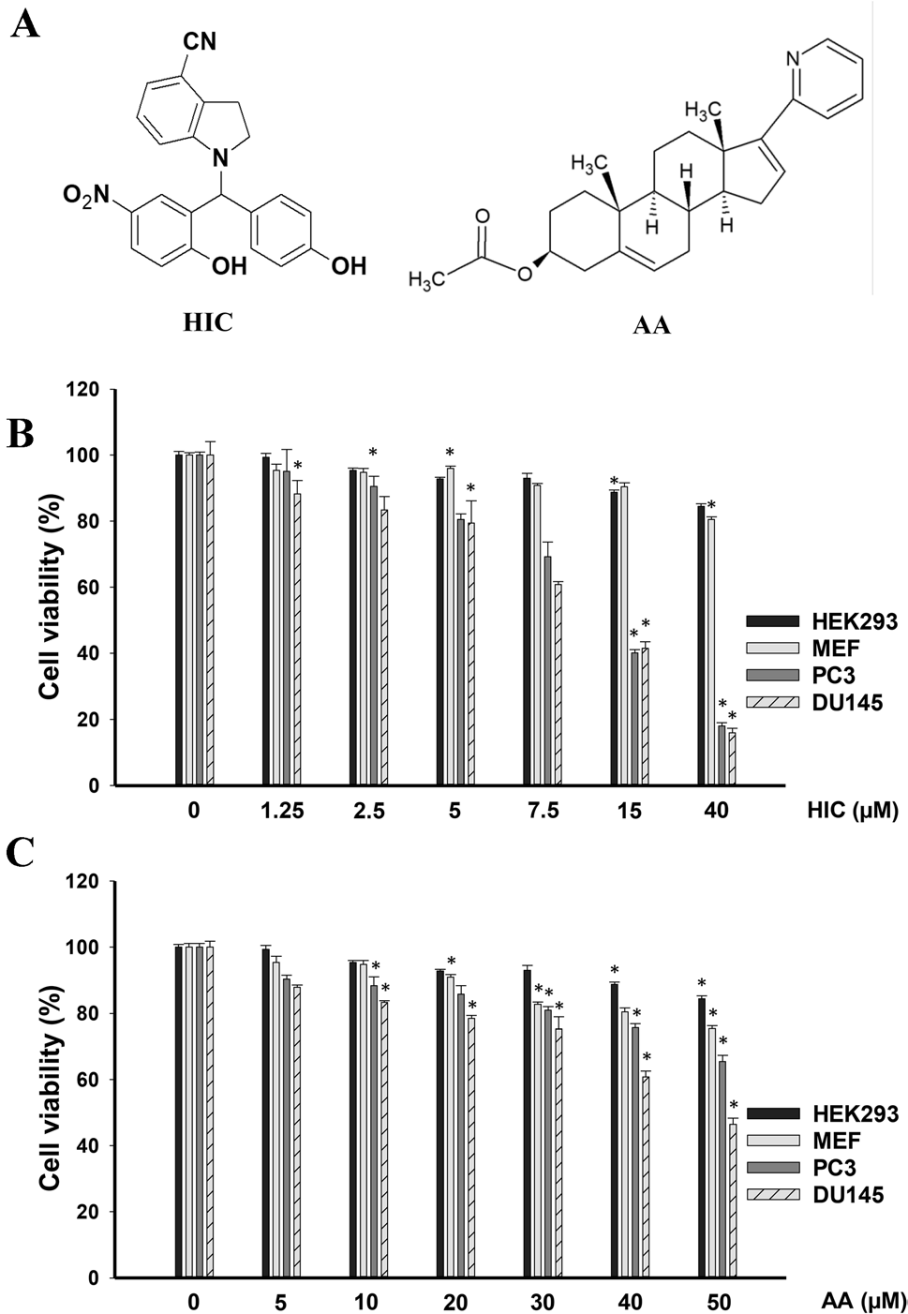
Sensitivity of PCa and noncancer cells to HIC and AA. Structure of HIC and AA (**A**). Percentage of cell viability with HIC treatment (**B**) and AA treatment (**C**) were determined using MTT assay. PC3, DU145, HEK293, and MEF cells were treated with increasing doses of HIC and AA for 48 h. Values are presented as the means ± SD of three biological experiments. *p < *0.05* relatives to DMSO-treated group

To assess the effective concentrations of HIC and AA (Fig. 1A) on cell death, PCa and non-cancer cells were incubated with increasing concentrations of HIC and AA for 48 h and cell viability was determined by MTT assay. Results show that HIC decreased cellular growth in a concentration-dependent manner compared to vehicle-treated cells (Fig. 1B). At a dosage of 40 μM HIC, PC3 and DU145 cells were inhibited to ~ 80% after 48 h treatment. At the same concentrations of HIC, the cell growth of HEK293 and MEF cells was similar to DMSO control groups (Fig. 1B). The IC_50_ values of HIC for PC3 and DU145 cells were determined as 15.98 μM and 15.64 μM, respectively. Conversely, AA showed lower inhibitory effects on the growth of PCa cells than HIC at 40 μM after 48 h treatment (Fig. 1C). The cell death was observed at about 34.6 ± 1.8% and 43.6 ± 1.9% in PC3 and DU145 cells at 40 μM AA treatment. These results are consistent with prior findings that AA had slight inhibitory effects on cell proliferation of PC3 and DU145 cells which have low expression of AR [22, 23]. Therefore, our results suggest that PC3 and DU145 cells are more sensitive to HIC than AA.

### HIC and AA cotreatment reduces cell growth in PCa cells

**Fig. 2:**
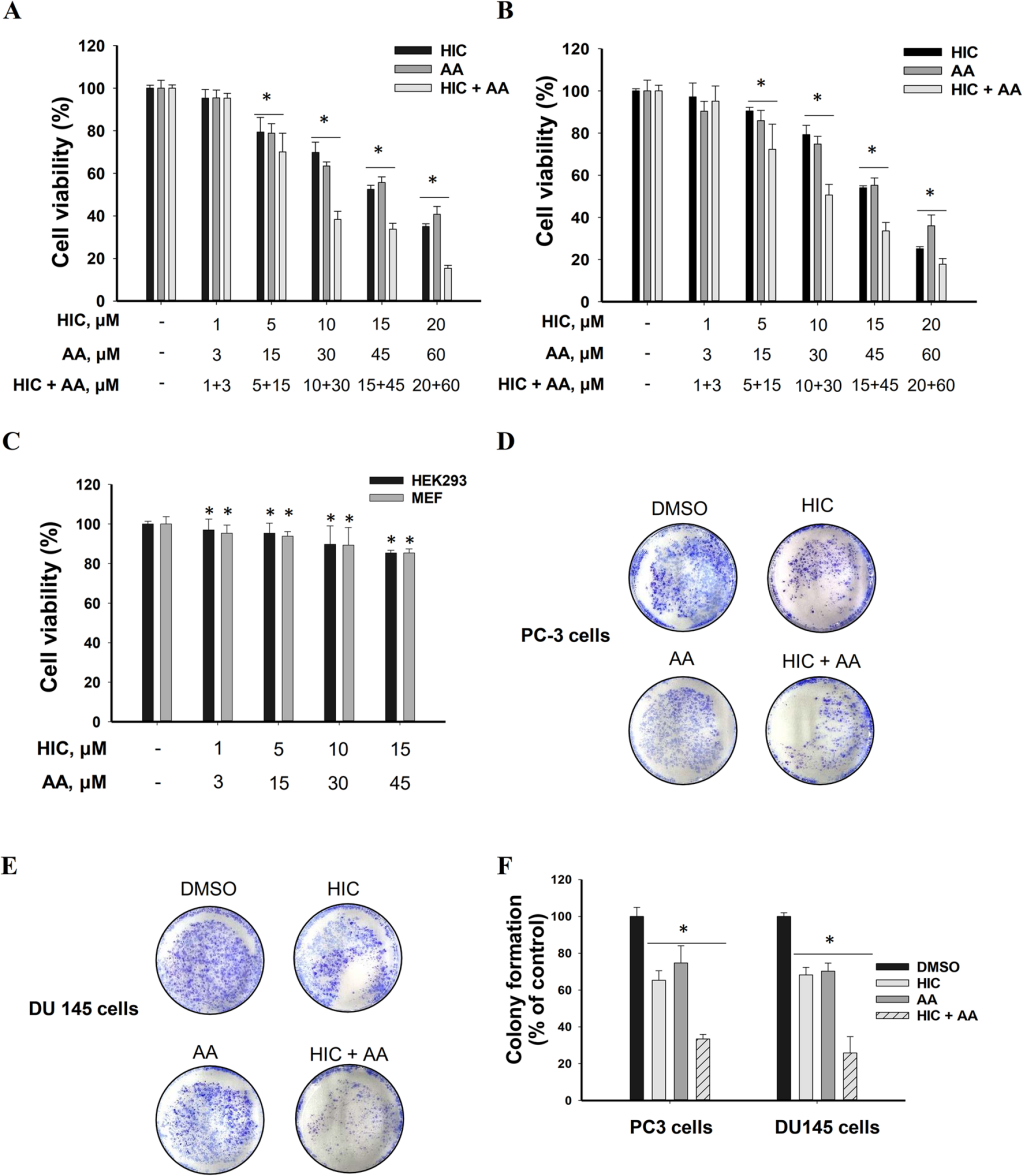
HIC and AA co-treatment reduces cell growth in PCa cells. Percentage of PC3 (**A**) and DU145 (**B**) cells viability in the presence of HIC and AA, alone or in combination with indicated concentrations. Percentage of cell viability for HEK293 and MEF cells with co-treated HIC and AA (**C**). Representative images of PC3 (**D**) and DU145 (**E**) cells treated with HIC, AA, or a combination of both in Clonogenic survival assay. The bar graph presented the colony formulation of PCa cells with HIC and AA treatment based on the normalization with DMSO groups (**F**). Data were analyzed by one-way ANOVA; *p < *0.05* relatives to DMSO-treated group

Next, to understand the effect of combined drugs treatment, PC3 and DU145 cells were treated with HIC and AA alone and the combination of HIC and AA (HIC + AA) at specific concentrations, as described in the methods section. Based on the sensitivity of HIC and AA on PC3 and DU145 cells, HIC and AA concentrations were adjusted to maintain a constant 1:3 ratio of HIC:AA for PC3 and DU145 cells. When HIC and AA were combined, a dramatic reduction in cell viability was observed (Fig. 2A, B). Cell death in both PC3 and DU145 cells ~ 50% was observed when in presence of 15 μM HIC or 45 μM AA (Fig. 2A, B). At an intermediate dosage of combined 10 μM HIC and 30 μM AA cell proliferation was inhibited more than 50% in both cell lines. These results identified that the co-treatment of HIC + AA induced stronger inhibitory effects on cell growth in PCa cells than using a single drug treatment. Subsequently, HEK 293 and MEF cells treated with the same concentrations of two drugs did not show a significant decrease in cell survival compared with vehicle groups (Fig. 2C). The synergistic effect of HIC and AA was analyzed based on CI values. Table 1 shows the CI values for DU145 and PC3 cells treated with the combination of HIC and AA. CI values less than 0.9, from 0.9 to 1.1, or more than 1.1 allow indicating the quantification of synergism, additive, or antagonism of two drugs, respectively. In addition, the combination of 10 μM HIC and 30 μM AA was less sensitive to the non-cancer cells survival (Fig. 2C) than for PCa cells (Fig. 2A, B). Therefore, these combinatorial concentrations were selected to perform further experiments.

**Table 1:** Combination index (CI) values for combined use of HIC and AA in PC3 and DU145 cells

Cell lines	HIC μM	AA μM	Effect %	CI	Cell lines	HIC μM	AA μM	Effect %	CI
PC3 cells	1	3	4.88	2.02	DU145 cells	1	3	6.71	1.89
	5	15	27.71	1.33		5	15	30.02	1.49
	10	30	49.43	1.11		10	30	61.63	0.85
	15	45	66.45	0.88		15	45	66.23	1.06
	20	60	82.23	0.57		20	60	84.69	0.53

To determine the anticancer effect of combined HIC and AA, the effects of both drugs on tumour cell clonogenicity in PC3 and DU145 cells were examined. Cells were treated with HIC and AA as described in the methods section. As shown in Fig. 2D, E, cotreatment with HIC and AA markedly inhibited PCa colony formation more than in single drugs treatment. In detail, PC3 colony formation was reduced by 34.7%, 25.3%, and 59.5% by HIC, AA, and HIC + AA combined treatments, respectively (Fig. 2F). The effect of HIC and AA cotreatment was 1.7 and 2.4 times more effective than HIC and AA alone. Similarly, the decrease of DU145 colonies was observed as 31.7%, 23.7%, and 60.1% by the presence of HIC, AA, and HIC + AA, respectively (Fig. 2F). The combined use of HIC and AA increased 1.5 to 2 times the effect of single AA in both PC3 and DU145 cells. These data purpose that the combinatorial treatment of HIC + AA can stop the colony formation. It is worth to suggest that the combinatorial treatment can stop the PCa cells division in vitro.

### Combination of HIC and AA inhibits the proliferation of PCa cells in a time-dependent manner

**Fig. 3:**
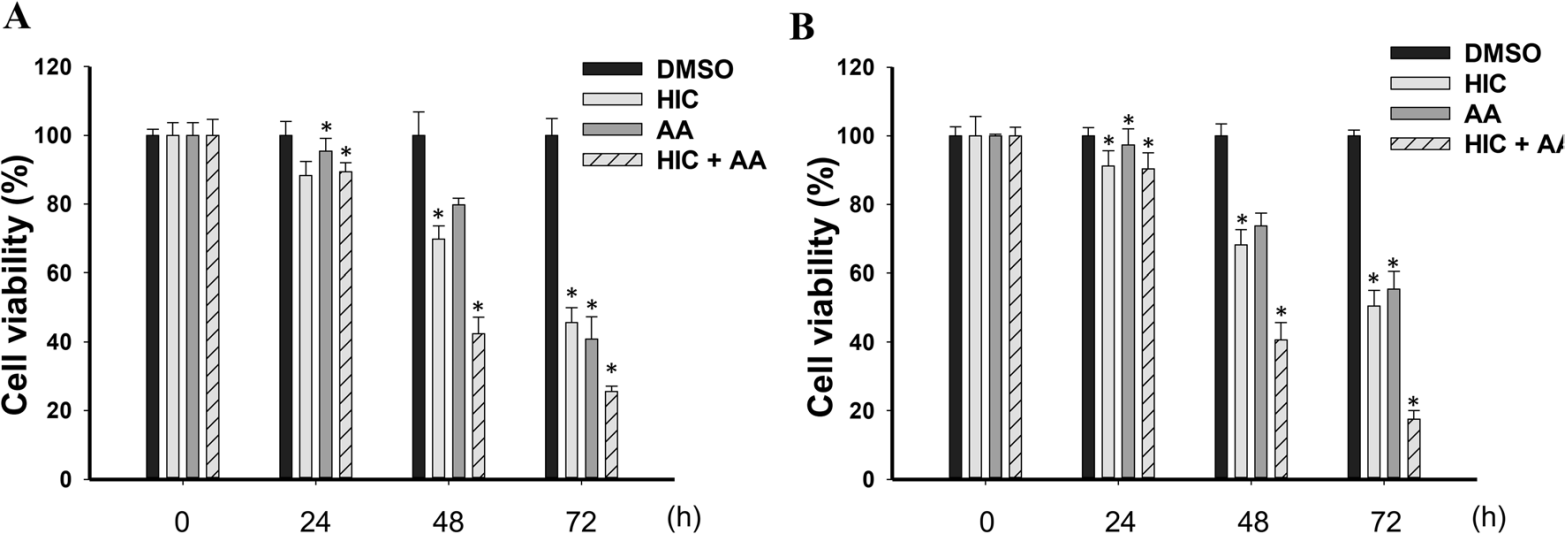
Combination of HIC and AA inhibits the proliferation of PCa cells in a time-dependent manner. Percentage of cell viability in **A** PC3 and **B** DU145 cells treated with HIC, AA, and HIC + AA combination for 24, 48, and 72 h. The percentage of cell survival was normalized to DMSO groups. The experiment was performed with n = 6. Statistical significance was considered with *p < *0.05*

Growth kinetics of cells treated with HIC, AA and HIC + AA were measured as described in the methods section to understand the effect of using combined drugs over time. As shown in Fig. 3A, the cell proliferation in treated PC3 cells was significantly lower in HIC and AA treatment than in the control group over time. The inhibition on the proliferation of PC3 cells was about 11.7%, 30.1%, and 54.4% by HIC, and about 4.6%, 20.1%, and 59.2% by AA treatments, at 24 h, 48 h, and 72 h respectively. However, a stronger inhibitory effect was observed in the combined use of HIC and AA groups after 24 h treatment. HIC and AA cotreatment induced 10.6%, 57.6%, and 74.4% of PC3 cell growth at 24 h, 48 h, and 72 h, respectively. Similarly, in DU145 cells, the cell proliferation was decreased from 50.5 to 97.3% progressively from 24 to 72 h on the treatment with HIC and AA. However, the impact of HIC and AA co-presence showed a reduced proliferation to 90.3%, 40.5%, and 17.5% at 24, 48, and 72 h, respectively. These findings suggest that the proliferation was inhibited on treatment with HIC + AA stronger than in single-drug treatments. In addition, HIC and AA could inhibit cancer cell proliferation by increasing the treatment time.

### Combinatorial effect of HIC and AA induces apoptosis through caspase 3/7 activation and ROS production

**Fig. 4:**
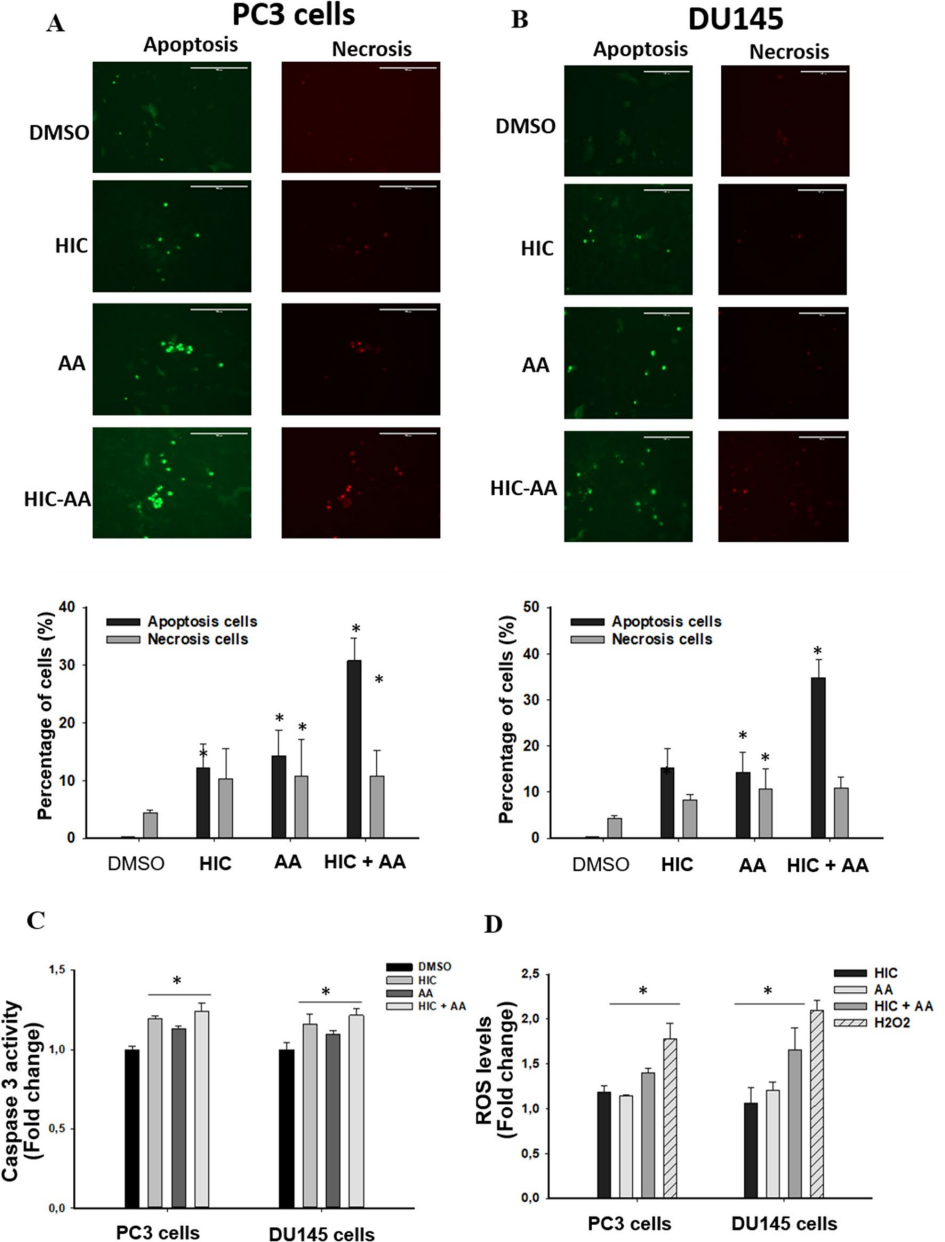
Combinatorial effect of HIC and AA induces apoptosis, caspase 3/7 activation, and ROS production. Representative images of PC3 (**A**) and DU145 (**B**) cells stained with DAPI/Annexin-V/PI in DMSO, HIC, AA, and cotreated drugs condition. Percentage of apoptotic and necrotic cells were presented in the corresponding conditions. **C** Fold changes of caspases 3/7 activity in PC3 and DU145 cells compared to DMSO groups. **D** Fold change of ROS production in PC3 and DU145 cells were calculated using fluorescence intensities based normalized against DMSO control. Biological and technical replicates were performed to analyze the results, with mean ± SD, *p < *0.05*, n = 6

To investigate whether synergistic loss of cell viability in PCa cells was related to the apoptotic process or not, we further examined the effect of drugs by Annexin V-affinity assay. Post 48 h of treatment, the fluorescent microscope images of PCa cells were captured to expose the presence of apoptotic (green) and necrotic (red) cells (Fig. 4). As shown in Fig. 4A, co-treatment of HIC and AA significantly increased the number of apoptotic cells (green) more than DMSO groups after 2 days of treatment. The increase of apoptotic cells in PC3 cells was observed ~ 12.2% and 14.2% by HIC and AA single treatment, respectively, whereas the combinatorial drugs induced 40.4% apoptotic population (Fig. 4A). Similarly, the induction of apoptotic responses in DU145 cells was 34.8% in the co-treatment of HIC and AA whereas 15.2% and 14.3% increases were determined in treatments with HIC and AA, respectively. These findings suggest that AA in combination with HIC induced apoptosis in PC3 and DU145 cells in agreement with the observed inhibitory effects on cell survival.

The activation of Caspase 3/7 is known as the crucial player in cell death and apoptosis [35]. To investigate the anticancer effect of HIC and AA, a Caspase 3/7 assay was performed in PC3 and DU145 cells. On treatment with the combination of HIC and AA, PC3 and DU145 cells have shown increasing fold changes of Caspase 3/7 (Fig. 4C). We noticed an increase in fold change of caspase 3/7 activity to 1.19 and 1.12 in HIC- and AA-treated PC3 cells, respectively, when compared to the vehicle group. Interestingly, the increase of Caspase 3/7 activity was observed in HIC and AA-cotreated PC3 cells with a fold change of about 1.24. Likewise, the activation of Caspase 3/7 in DU145 cells also increased to 1.15- and 1.09-fold change on treatment with HIC and AA whereas HIC + AA showed 1.21 caspase 3/sevenfold change. The difference in the fold change of treated and untreated groups was considered as a statistical analysis through ANOVA test with *p* < *0.05*. These findings showed that the combined treatment with HIC and AA, induced more caspase activity than treatment with one of the single compounds. Moreover, HIC and AA could induce apoptosis through the Caspase 3/7 dependent signaling pathway. It is noted that caspase-3 play a crucial role in the apoptosis process in various cell lines, which is primarily responsible for the cleavage of poly(ADP-ribose) polymerase (PARP) during cell death. In our previous study [36], we have reported the total gene expression profile of PC3 and DU145 cells treated with HIC compound. We found the downregulation of PARP10 and PARP12 expression in both the cell lines. Furthermore, PARP1 and PARP14 was also downregulated in PC3 cells, while PARP9 was downregulated in DU145 cells. These observations further support that caspase 3 is activated upon the treatment of HIC compound.” The differential expression of PARP is plotted as graph and presented as a supplementary Fig. 1.

Accumulation of ROS at mitochondria is one of the apoptotic mechanisms in the intrinsic cell death pathway [37, 38]. A high level of ROS might damage proteins, nucleic acid, and result in oxidative stress and cellular dysfunctions [39]. In addition, ROS is known as a capable stimulator inducing cell cycle arrest and cell death in cancer therapeutics [40]. In order to investigate the effect of HIC and AA combination on PCa via ROS productivity, PC3 and DU145 cells were treated with HIC, AA, and H_2_O_2_ (positive control). As shown in Fig. 4D, the fold change of ROS production increased in the presence of HIC, AA, and H_2_O_2_ in both cell lines. The fold change of ROS was increased ~ 1.2 in both HIC- or AA-treated PCa cells. HIC and AA co-treatment increased the fold change of ROS with 1.4 and 1.6 in PC3 and DU145 cells, respectively. Therefore, ROS productivity was noticeably higher in HIC + AA-treated cells than in HIC- and AA-treated cells.

### Combined use of HIC and AA suppresses the G1 phase of the PCa cell cycle

**Fig. 5:**
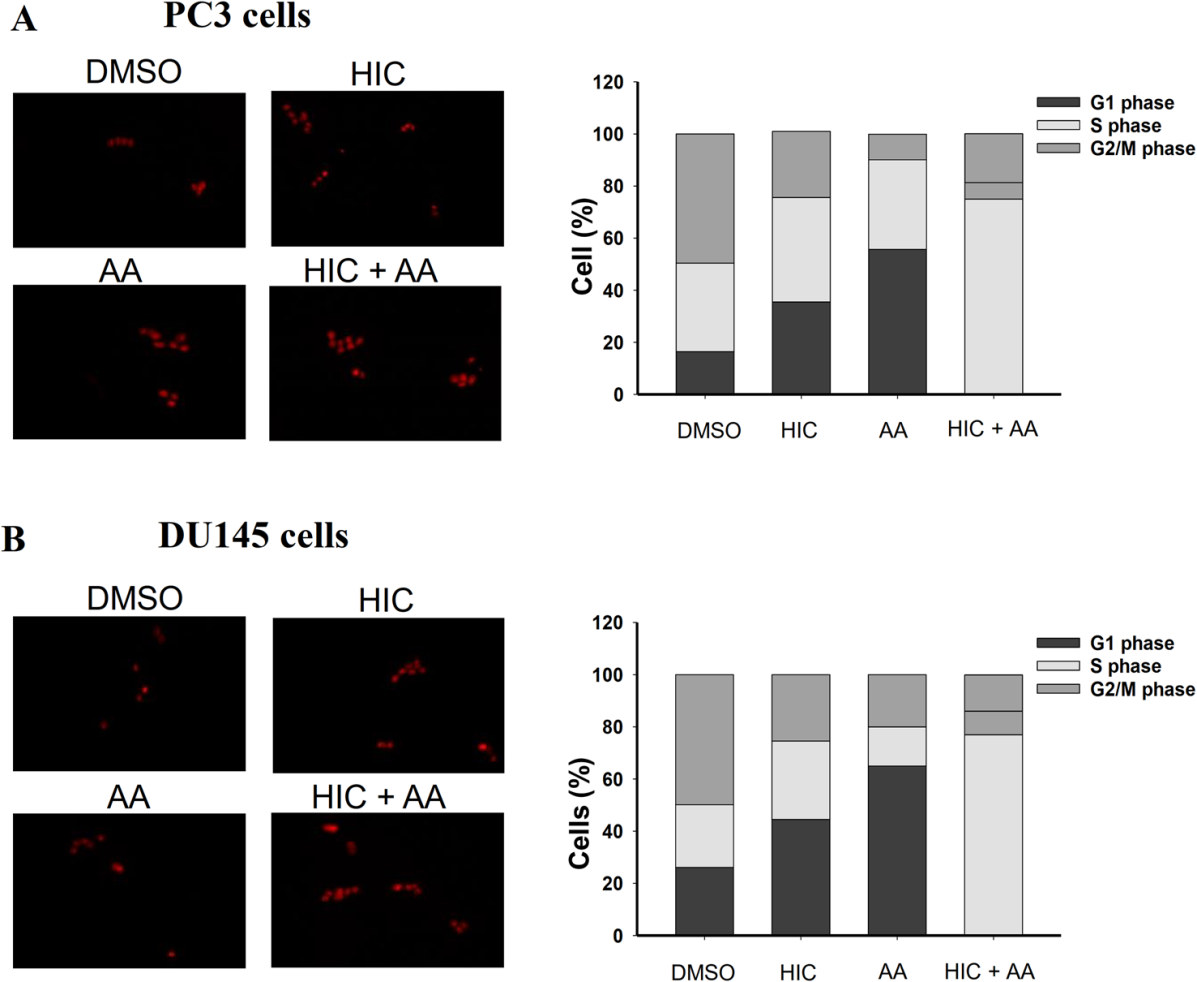
Combination of HIC and AA suppresses the G1 phase of the PCa cell cycle. PC3 and DU145 cells were treated with HIC and AA alone or in combination for 48 h. Cells were fixed and stained with PI. Microscopic images of PC3- (**A**) and DU145- (**B**) treated cells were captured and analyzed to detect cell-cycle distribution. The percentage of cells in each phase was calculated and presented. Data is shown as mean ± SD, *p < *0.05*, n = 6

Apoptosis is the major cellular response that can regulate cell cycle arrest and induce cell death [41, 42]. To evaluate whether the combination of HIC and AA induces cell cycle distribution in PCa cells, cell cycle analysis was examined as described in the methods section. Fig. 5A, B exemplify fluorescence images of DNA content in each cell phase, treated by selected compounds. The cells were segmented to detect the distribution of each phase under different conditions. The proportion of the G1 phase of PC3 cells was increased after treatment with HIC and AA by 35.5% and 55.7%, respectively, compared to the vehicle group with 16.4% for the G1 phase. Interestingly the combined use of HIC with AA induced the highest G1 proportion to 75.1% (Fig. 5A). The transition of S phase of PC3 cells was not significantly different among vehicle, HIC, and AA treatment, about 34.0%, 40.1%, and 34.4%, respectively. Interestingly, the distribution of G1 phase in DU145 cells was observed as the same as in PC3 cells. A higher percentage of the G1 phase, 77.1%, was observed in the combinatorial treatment of HIC and AA. The proportion of G1 phase in DU145 cells treated with HIC and AA was calculated as 44.5% and 60.5%, respectively. Furthermore, in the S phase of DU145 cells, the percentage of the population in HIC, AA, and HIC + AA, and vehicle-treated cells was 30.0%, 15.0%, 9.0%, and 24.0%, respectively. Notably, a higher fraction of G1 phase arrest in both PC3 and DU145 cells was observed in HIC and AA co-treated groups. Taken together, the results suggested that the combined treatment of HIC and AA arrested the PCa cells at G1 proliferation phases, leading to higher suppression than the treatments with HIC and AA alone.

### Metastatic activity of PCa cells was inhibited by treatment with HIC and AA

**Fig. 6:**
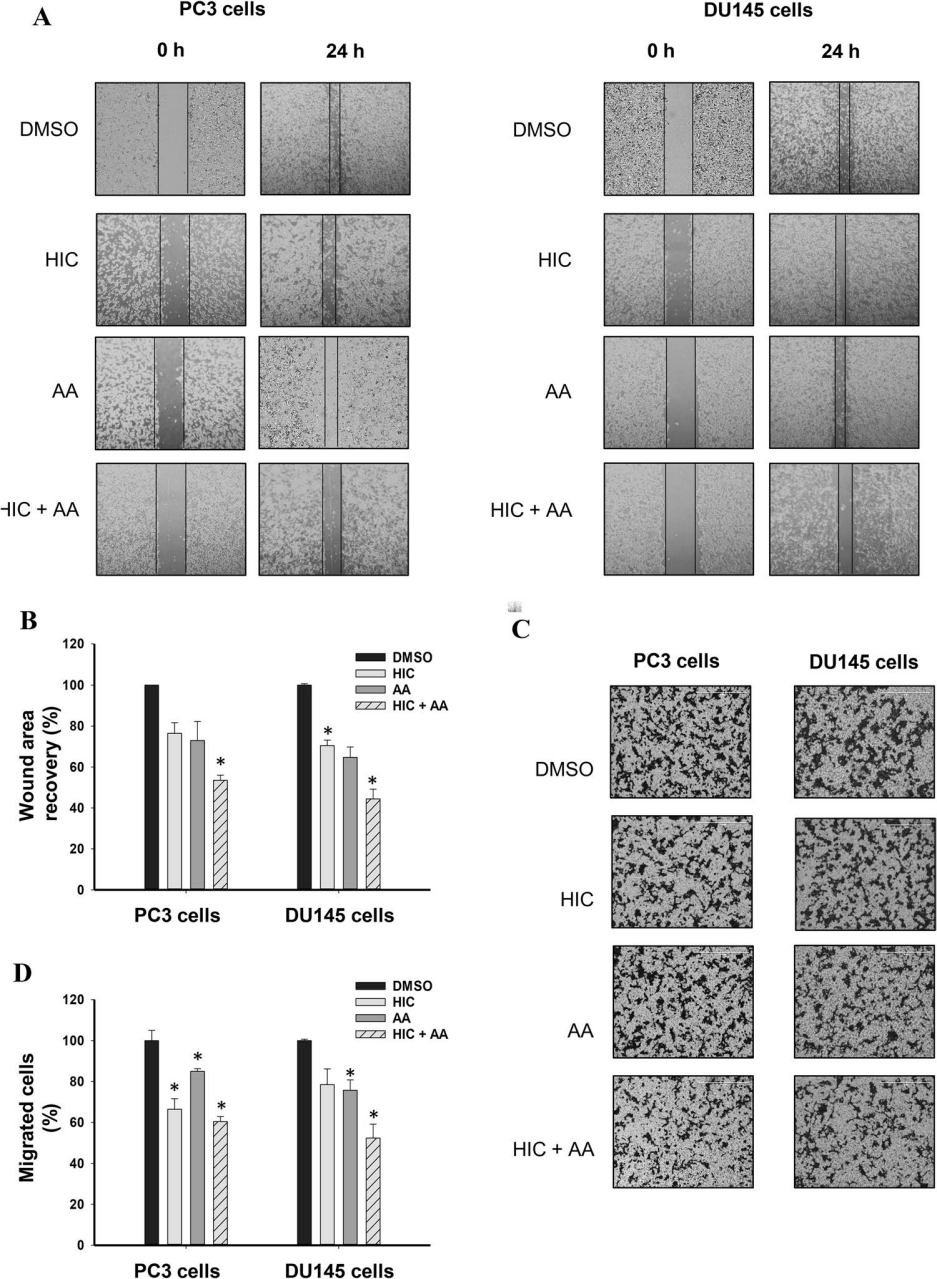
Inhibition of metastatic properties of PCa cells. **A** The wounds of PC3 and DU145 cells were created using a scratcher, and then cells were incubated with HIC, AA, alone or a combination of both. Representative images were captured at 0 and 24 h. A mark in these images was placed to locate the same area on the scratch. Percentage of relative wound closure was shown as a bar graph. **B** Invaded cells were captured under the microscope. The bar graph presented is the percentage of cell invasion based on DMSO groups. Experiments were repeated three times with technical repeats, n = 6, *p < *0.05*, ANOVA test

The cell migration and invasion are known as important characteristics of malignant tumour cells, thus inhibiting migration and invasion of cells are considered as crucial targets in developing new anticancer therapeutics [43, 44]. Here we performed the cell migration and invasion assay to investigate the effect of HIC and/or AA on PC3 and DU145 cells. As shown in Fig. 6A, the PCa cells were scratched and treated with drugs. The invaded areas of PCa cells after HIC + AA treatment decreased steadily after 12 h of incubation whilst it increased over time after DMSO treatment. 24 h of post-treatment, the invaded areas of PC3 cells decreased to 23.5%, 27.1%, and 46.4% by HIC, AA, and HIC + AA, respectively. Notably, a similar pattern was also observed in DU145 cells. The percentage of migrated cells in DU145 cells was reduced to 29.5% by HIC, 35.2% by AA, and 55.5% by HIC + AA treatment. In addition, to further explore the anti-metastasis effect on the invaded cells, we performed the invasion assay with HIC and AA co-treatment. PC3 and DU145 cells were treated with HIC and AA and then measured the invaded activity via Matrigel-coated transwell after 24 h treatment. As shown in Fig. 6B, cell invasion decreased in both cells when drugs were present. A significant decrease caused by HIC and AA co-treatment was observed in DU145 cells. In detail, the invaded cells in DU145 cells were inhibited to 78.5%, 75.7%, and 52.4% by HIC, AA, and HIC + AA treatments, respectively (Fig. 6B). These results demonstrate that the combination of both HIC and AA potentially inhibits cell migration and invasion of PCa cells.

## Discussion

In the present study, we characterized the pharmacological activity of HIC, P2Y1 receptor agonist, in androgen-independent cancer cell lines models PC-3 and DU145. Former studies concerning HI, have served as proof of principle: PC-3 and DU145 cell growth inhibition was induced by HIC were the compound act as a p53 stabilizer in prostate cancer cells, however, the detailed molecular mechanism of HIC in combination with any clinical drugs are yet to be investigated [30]. Here, we investigated the potential mechanism involved in the inhibition of cell growth in AR-negative-cell lines, PC3 and DU145, by AA and HIC co-treatments. Our results suggest that these cell lines were sensitive to HIC at the IC_50_ range of 15–18 μM after 48 h treatment. The metastatic cell lines PC3 and DU145 which are both AR low and represent castrate-resistant metastatic disease, are equally responsive to HIC. AR-negative PC3 and DU145 cells exhibited comparably growth inhibition responses to the presence of AA.

The applied AA concentration of 40 μM induced ~ 50% cell death after 48 h treatment. AA was minimally effective in killing PC3 and DU145 cells at the concentration utilized, but the combination of both compounds results in synergistic-induced loss of cell viability. Moreover, normal cell lines are less sensitive to the combined effects of HIC and AA than PCa cells in the same conditions. Concerning the apoptosis produced by the combination of HIC and AA, we showed that Caspase 3/7 activity and ROS formulation increased due to the synergic effect of the two drugs. One possible mechanism of cell death is cell cycle arrest. The cell phases arrested by AA and HIC in these cells have not been reported. Here, we found that the combination between HIC and AA suppressed the G1 phase in both cell lines.

Besides the numerous AR signaling events in PCa cells, chemotherapy widely exerts its anticancer effect by triggering apoptotic mechanisms of tumour cells [45]. A primary regulator of apoptosis is the tumour suppressor p53, which plays a key role in cell cycle control, genomic stability, and apoptosis [46]. Increased DNA fragmentation as well as downregulation of the cell survival factor surviving also confirm the involment of execution of apoptotic mechanisms. Further, increased level of the cell cycle inhibitor p21 and the effector caspase3 also confirms the role of apoptosis ativation [47], 48. From our previous studies, HIC was known to induce the stability of p53 and regulate p21 signaling in PCa cells. The decrease of mRNA levels was observed in *CDK2*, *Cyclin E*, *Cyclin A*, *CDK4* in the incubation of HIC in PC3 and DU145 cells [30]. Interestingly, DNA fragment was determined as one factor in the HIC downregulation mechanism. Moreover, the levels of *BAX*, *Caspase3*, *p21*, and *Survivin* also increased in the presence of 30 μM of AA after 48 h in PC3 cells [23]. In this work, our results showed the increase of Caspase3/7 activity and ROS production with the combined use of HIC and AA. Notably, the G1 phase was arrested by the synergic effect of HIC and AA after 48 h. Taken together, our observations suggest that AA and HIC could mediate the cell death in PC3 and DU145 cells through the activation of apoptosis via p53, p21 signaling. AR signal is not the only rationale to explain the anticancer activity of AA in PCa cells. In addition, the presence of HIC + AA potentially inhibited cell proliferation. Therefore, the combination of HIC and AA would be considered as a promising agent in the treatment of AR-negative PCa cells. In summary, the activation of the P2Y1 receptor by HIC and its combination with AA demonstrated that the HIC might be an attractive agent for the treatment of prostate cancer.

## Conclusion

In summary, our results show that the HIC and AA induce apoptosis-mediated cell death through the activation of Caspase3/7 and ROS production. The synergistic effect of HIC and AA affects prostate cancer cells proliferation in a time-dependent manner, and their migration, invasion, colony-forming ability, and cell cycle progression in the G1 phase. More importantly, the combination of two compounds, a P2Y1 receptor agonist and an androgen receptor inhibitor, is found to be a potential combinatorial drug to overcome prostate cancer.

## Supplementary Information

Below is the link to the electronic supplementary material.

Supplementary file1 (DOCX 120 KB)

## Data Availability

The datasets generated analysed during the current study are available from the corresponding author on reasonable request.

## Abbreviations

HIC: 1 (1-(2-Hydroxy-5-nitrophenyl) (4-hydroxyphenyl) methyl)indoline-4-carbonitrile)
P2Y1R: Purinergic G-protein coupled receptor
AR: Androgen receptor
PCa: Prostate cancer
GPCRs: G protein-coupled receptors
PBS: Phosphate-buffered saline
DMSO: Diluted in dimethyl sulfoxide
MTT: 3-(4,5-Dimethylthiazol-2-yl)-2,5-Diphenyltetrazolium Bromide
DAPI: 4′,6-Diamidino-2-phenylindole
AA: Abiraterone acetate
PI: Propidium iodide
