# Supplementary material for: P2Y1 agonist HIC in combination with androgen receptor inhibitor abiraterone acetate impairs cell growth of prostate cancer
Source: Apoptosis. 2022 Feb 7;27(3-4):283–95. doi: 10.1007/s10495-022-01716-1 (PMC8940814; doi:10.1007/s10495-022-01716-1)
Supplement: Supplementary file 1 — Supplementary file1 (DOCX 120 KB) [file 10495_2022_1716_MOESM1_ESM.docx]

**Supplementary document**

**P2Y1 agonist HIC in combination with androgen receptor inhibitor** **Abiraterone acetate impairs cell growth of prostate cancer**

Hien Thi Thu Le^1^, Akshaya Murugesan^1,2^, Nuno R. Candeias^3,4^, Thiyagarajan Ramesh^5^, Olli Yli-Harja^6,7^, and Meenakshisundaram Kandhavelu^1*^

^1^Molecular Signaling Lab, Faculty of Medicine and Health Technology, Tampere University and BioMediTech, P.O.Box 553, 33101 Tampere, Finland.

^2^Department of Biotechnology, Lady Doak College, Thallakulam, Madurai – 625002, India.

^3^Faculty of Engineering and Natural Sciences, Tampere University, Korkeakoulunkatu 8, 33101 Tampere, Finland

^4^LAQV-REQUIMTE, Department of Chemistry, University of Aveiro, 3810-193, Aveiro, Portugal

*^5^*Department of Basic Medical Sciences, College of Medicine, Prince Sattam Bin Abdulaziz University, Al-Kharj, 11942, Kingdom of Saudi Arabia

^6^Computational Systems Biology Research Group, Faculty of Medicine and Health Technology and BioMediTech, Tampere University, P.O.Box 553, 33101 Tampere, Finland.

^7^Institute for Systems Biology, 1441N 34th Street, Seattle, WA 98103-8904, USA

*Corresponding author: [Meenakshisundaram.kandhavelu@tuni.fi](mailto:Meenakshisundaram.kandhavelu@tuni.fi)

**Supplementary Figure 1:**


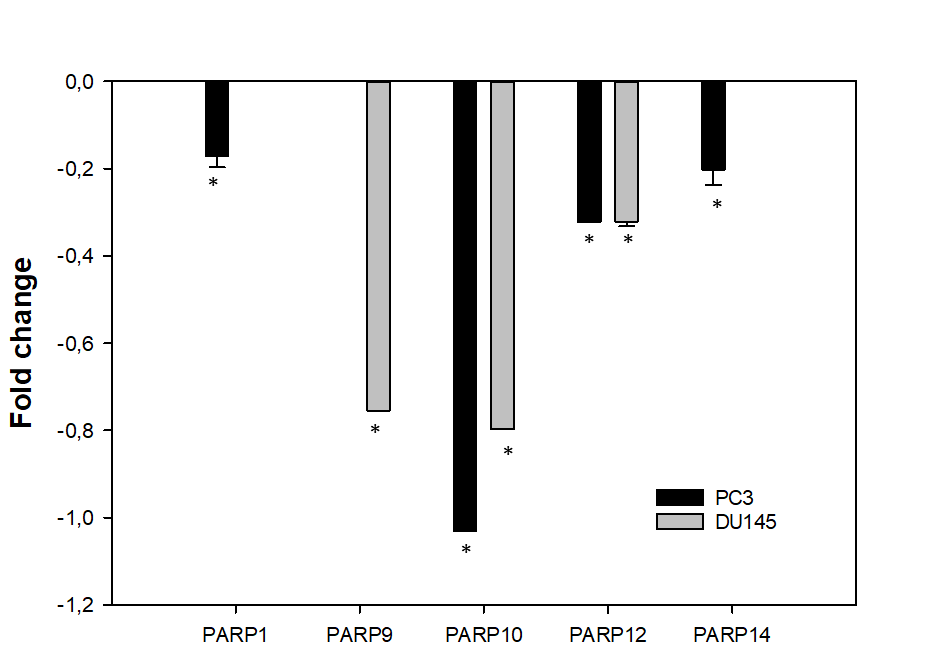


**SF1, Inhibition of Poly(ADP-Ribose) Polymerase by HIC treatment:** RNAseq analysis was performed to study the effect of HIC in PC3 and DU145 cells at the transcriptome level (Thu Le et al., 2021). The detailed description about the treatment, mRNA extraction and Illumina sequencing and bioinformatics analysis protocol were already reported (Le et al., 2021) by our group. The graph shows the differential expression of PARP family genes involved in caspase 3 upon HIC treatment in PC3 and DU145 cells. Downregulation of PARP in both the cell lines was observed. The expression of differentially expressed genes was considered significant with the *p < 0.05.

**Supplementary Figure 2:**

**
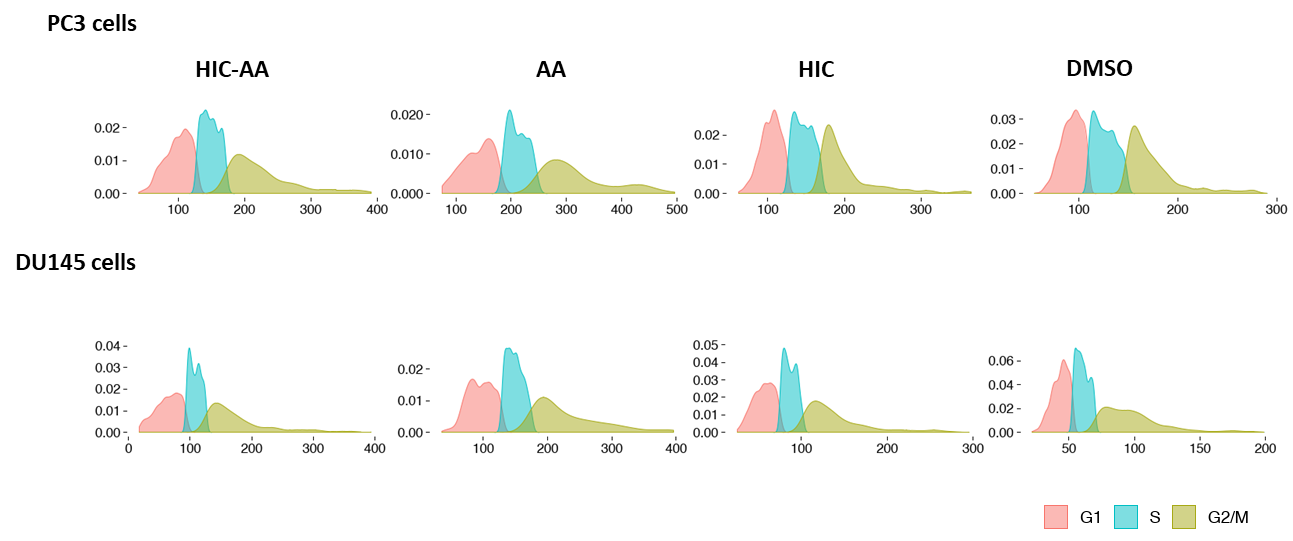
**

**SF2, Distribution of cells in different phases of cell cycle**: Graph shows the cell cycle phase of PC3 and DU145 cells treated with DMSO, HIC, AA and HIC+AA.

**Reference:**

Thu Le, H.T., Murugesan, A., Candeias, N.R., Yli-Harja, O., Kandhavelu, M., 2021. Functional characterization of HIC, a P2Y1 agonist, as a p53 stabilizer for prostate cancer cell death induction. Future Med. Chem. https://doi.org/10.4155/fmc-2021-0159
